# Dietary Patterns and Cerebral Glucose Metabolism in Older Adults: Findings from the Western Australian Memory Study

**DOI:** 10.3390/nu18071136

**Published:** 2026-04-01

**Authors:** Carolina B. Castro, Samantha L. Gardener, Farzana Jahan, Juliana Chen, Belinda M. Brown, Ruey L. Loo, Kevin Taddei, Stephanie R. Rainey-Smith, Michael Weinborn, Ana Caroline R. dos Reis, Shipra Verma, Nick Carrigan, Charles Inderjeeth, Vincent Doré, Manohar L. Garg, Ralph N. Martins, Hamid R. Sohrabi

**Affiliations:** 1 Centre for Healthy Ageing, Health Futures Institute, Murdoch University, Murdoch, WA 6150, Australia; farzana.jahan@murdoch.edu.au (F.J.); belinda.brown@murdoch.edu.au (B.M.B.); stephanie.raineysmith@murdoch.edu.au (S.R.R.-S.); 2 Alzheimer’s Research Australia, Nedlands, WA 6009, Australia; samantha.gardener@uwa.edu.au (S.L.G.); ralph.martins@mq.edu.au (R.N.M.); 3 School of Psychology, Murdoch University, Murdoch, WA 6150, Australia; 4 Macquarie Medical School, Faculty of Medicine and Health Sciences, Macquarie University, Sydney, NSW 2109, Australia; manohar.garg@mq.edu.au; 5 The UWA Medical School, The University of Western Australia, Perth, WA 6009, Australia; 6 School of Mathematics, Statistics, Chemistry and Physics, College of Science, Technology, Engineering and Mathematics, Murdoch University, Murdoch, WA 6150, Australia; 7 The University of Sydney, Faculty of Medicine and Health, Camperdown, NSW 2006, Australia; juliana.chen@sydney.edu.au; 8 Lifestyle Approaches Towards Cognitive Health Research Group, Murdoch University, Murdoch, WA 6150, Australia; 9 Australian National Phenome Centre and Centre for Computational and Systems Medicine, Health Futures Institute, Murdoch University, Perth, WA 6150, Australia; rueyleng.loo@murdoch.edu.au; 10 School of Psychological Science, The University of Western Australia, Crawley, WA 6009, Australia; 11 State University of Bahia (UNEB), Department of Life Sciences, Salvador 41150-000, BA, Brazil; 12 Department of Nuclear Medicine, PET CT and Radionuclide Therapy, Fiona Stanley Hospital, Murdoch, WA 6150, Australia; 13 Department of Geriatric Medicine, Fiona Stanley Hospital, Murdoch, WA 6150, Australia; 14 School of Medicine, The University of Western Australia, Crawley, WA 6009, Australia; 15 Gerontorheumatology, Sir Charles Gairdner Osborne Park Health Care Group, Perth, WA 6009, Australia; 16 Health and Biosecurity Flagship, Australian eHealth Research Centre, CSIRO, Parkville, VIC 3052, Australia; 17 Department of Molecular Imaging, Austin Health, Heidelberg, VIC 3084, Australia

**Keywords:** FDG PET, glucose metabolism, Alzheimer’s disease, Western Australian Memory Study, dietary patterns, Western Diet, Prudent Diet

## Abstract

Alzheimer’s disease (AD) is characterized by significant reductions in glucose metabolism, reflecting underlying synaptic dysfunction, correlating with cognitive decline. We aimed to explore the impact of dietary patterns on the change in glucose metabolism. Methods: This longitudinal, prospective study included 132 community-dwelling older adults without a diagnosed dementia history enrolled in the Western Australian Memory Study (WAMS). Participants completed a food frequency questionnaire at baseline and underwent [^18^F]-Fluorodeoxyglucose positron emission tomography (FDG-PET) imaging at baseline and at up to two follow-up assessments scheduled approximately 18 months apart, over a maximum follow-up period of 43 months. Principal component analysis yielded two dietary patterns—named Western Diet and Prudent Diet. Linear mixed-effect models evaluated the association between dietary adherence and glucose metabolism, including potential confounders. Analysis was repeated stratified by sex. Results: Adherence to a Western Diet, characterized by high sugars and saturated fats, was associated with faster decline in glucose metabolism in the left fusiform gyrus (β = −0.00062; SE = 0.00025; FDR-adjusted *p* = 0.043), neocortex (β = −0.00063; SE = 0.00026; FDR-adjusted *p* = 0.047), left ventrolateral prefrontal (β = −0.00083; SE = 0.00032; FDR-adjusted *p* = 0.045 and inferior parietal region (β = −0.00344; SE = 0.00129; FDR-adjusted *p* = 0.033) in females. A Prudent Diet, characterized by a high intake of fruits, vegetables, and whole grains, showed no significant effects. Conclusions: Our study highlights the following: (a) The potential detrimental impact of a Western Diet on brain glucose metabolism, particularly for females, who are at higher risk for AD. The decline was observed in regions essential for cognitive functions, including visual processing and facial recognition, emphasizing the role of diet in brain health. (b) No significant associations were observed between adherence to a Prudent dietary pattern and changes in glucose metabolism.

## 1. Introduction

Presently, more than 55 million individuals worldwide have a dementia diagnosis [1,2], leading to considerable economic and societal burden [3]. As the population ages, dementia cases are projected to rise to 152.8 million cases in 2050 [1]. Alzheimer’s disease (AD) is the most prevalent form of dementia, characterized by the accumulation of hyperphosphorylated tau within neurons and abnormal deposition of beta-amyloid (Aβ) in the form of extracellular plaques and deposits in blood vessel walls [4]. Dietary modifications, particularly those promoting nutrient-rich, antioxidant, and anti-inflammatory foods, have been identified as a potential preventative strategy to mitigate the risk of AD and its progression [5].

Imaging biomarkers, such as [^18^F]-Fluorodeoxyglucose positron emission tomography (FDG-PET), have shown promise in improving the accuracy of AD diagnosis and monitoring [6]. FDG-PET measures the “regional cerebral metabolic rate of glucose” (rCMRglc), which reflects the functional activity of brain regions. Research shows significant reductions in glucose metabolism in individuals with AD, particularly in brain regions such as the temporal, posterior cingulate, and inferior parietal cortex, as measured by FDG-PET [6]. Such hypometabolism reflects the underlying synaptic dysfunction and is a consistent biomarker in AD patients, correlating with cognitive decline [6,7]. While most neurodegenerative processes exhibit FDG-PET abnormalities, the topological pattern of glucose hypometabolism can aid in differential diagnosis, with the location and intensity of hypometabolism useful for identifying neurodegenerative type and severity. Studies using FDG-PET have consistently observed hypometabolism in these regions, highlighting glucose metabolism as an important biomarker for differential diagnosis of neurodegenerative processes [8].

Recent evidence suggests that sex differences in cerebral glucose metabolism may contribute to cognitive resilience during the early stages of AD. In a cross-sectional study, Sundermann et al. observed that females exhibited higher FDG-PET brain glucose metabolism than males among those with mild-to-moderate pathology burden (n = 1259; 56.5% male), particularly in the parietal and temporal cortices [9]. Importantly, this female metabolic advantage was associated with better global cognition, lower dementia severity, and superior executive function. However, as pathology progressed to more advanced stages, the female advantage in brain metabolism and cognitive function was eliminated, suggesting a higher metabolic and cognitive decline trajectory in females compared to males. These findings imply that higher brain energy utilization in females temporarily compensates for AD pathology, providing resilience against early cognitive deterioration. However, this reserve becomes insufficient as disease burden becomes severe, contributing to the more rapid decline observed in females in later stages of AD [9].

FDG-PET has been used to study the relationship between glucose metabolism and cognitive function in ageing populations. Gardener et al. found that glucose metabolism in the left hippocampus and right amygdala was positively associated with immediate recall of verbal logical memory in community-dwelling older adults [10]. However, there was no significant association between glucose metabolism and visual memory performance.

Emerging research suggests that diet may influence brain glucose metabolism. Studies show that Western-type dietary patterns, characterized by high intake of saturated fats and refined carbohydrates, can lead to insulin resistance, oxidative stress, and chronic inflammation, all of which impair glucose transport and utilization in the brain [11]. This disruption in glucose metabolism may reduce synaptic activity and contribute to the accumulation of Aβ. To our knowledge, no published studies have analyzed the association between a Western-type dietary pattern and glucose uptake in the brain in human cohorts. However, rodent studies have shown that 12 weeks of high-fat diet consumption decreases whole-brain glucose metabolism, as measured by FDG-PET, and impairs spatial memory retrieval and working memory in male rats but not in females [11]. These deficits are associated with reduced hippocampal expression of glucose transporter 3 (GLUT3), insulin receptor substrate 2 (IRS-2), and insulin-degrading enzyme (IDE), with the authors proposing the mechanism behind the dysregulation of brain glucose metabolism and cognitive performance involves disrupted insulin signalling pathways, leading to impaired glucose transport and energy metabolism in the hippocampus, a region critical for memory processing [11]. In addition, a recent study by Poxleitner et al. found that long-term consumption of a Western Diet in a mouse model of AD (APPPS1) disrupts FDG-PET-measured cerebral glucose and fatty acid metabolism, independent of Aβ pathology [12]. The study highlighted alterations in the brain-liver-fat axis and increased adaptive immune responses, with a notable involvement of cytotoxic T cells in brain inflammation. These findings suggest that diet-induced metabolic dysregulation may exacerbate neurodegenerative processes by disrupting peripheral and central metabolic homeostasis.

Conversely, the Prudent Diet, which emphasizes the consumption of whole grains, vegetables, fruits, lean proteins, and healthy fats [13], could be hypothesized to enhance cerebral glucose metabolism. However, to our knowledge, such a relationship has not been previously examined. The Prudent Diet is rich in antioxidants, polyunsaturated fatty acids (PUFAs), and dietary fibres, all of which have neuroprotective properties [13,14]. Antioxidants mitigate oxidative stress, while PUFAs improve insulin sensitivity and neuronal membrane fluidity [13,14]. Significant gaps remain in understanding the effects of dietary patterns, such as the Western and Prudent Diets, on cerebral glucose metabolism and how these effects may differ by sex [15].

This study aimed to explore the impact of two ‘a posteriori’ dietary patterns—the Western Diet (an ‘unhealthy’ dietary pattern), and the Prudent Diet (a ‘healthy’ dietary pattern)—on change in glucose metabolism as measured using FDG-PET at baseline and at up to two follow-up assessments scheduled approximately 18 months apart, over a maximum follow-up period of 43 months. The investigation was conducted on a well-defined cohort of older adults drawn from the Western Australia Memory Study (WAMS). The study was supported by an NHMRC grant (#324100; Molecular and Neuropsychological Predictive Markers of Cognitive Decline) and conducted under the Australian Alzheimer’s Research Foundation.

## 2. Methods

### 2.1. Participants

The WAMS is a longitudinal, community-based cohort initiated between 2006 and 2009 to investigate the molecular and neuropsychological predictors of cognitive decline. Exclusion criteria included clinically uncontrolled psychiatric disorders—including major depressive disorders, anxiety disorders, or schizophrenia—and a history of stroke or other neurological diseases (e.g., dementia of any type, Parkinson’s disease) affecting cognitive function. Participants were included (n = 132) if, at baseline, they (i) completed FDG-PET (Discovery 710 PET scanner, GE Healthcare, Preston, UK); (ii) completed the Cancer Council of Victoria Food Frequency Questionnaire (CCVFFQ), and (iii) donated a blood sample for genotyping.

### 2.2. Dietary Assessment

All participants completed the CCVFFQ [16,17], which assesses consumption of 74 dietary items and provides a comprehensive overview of participants’ regular diet over the past 12 months. The food composition data used to calculate daily nutrient intake are from the Nutrient Tables for use in Australia 1995 (NUTTAB95) [18].

#### Western and Prudent Dietary Patterns

For this study, 101 food and drink items were classified into 33 pre-defined food groups, minimizing within-person variations in intakes of individual foods. Factor analysis (principal components) was conducted to derive dietary patterns [19]. Food groups were entered as weight in grams per day. The factors were rotated using a varimax procedure to yield non-correlated factors, thereby facilitating factor interpretability. Components were retained based on eigenvalues > 1.25, inspection of the scree plot, Scree tests [19], and interpretability of the derived factors. A slightly more conservative eigenvalue threshold was applied to reduce over-extraction and improve the stability of dietary patterns. A cut-off of 0.30 was used to determine factor loadings included in each pattern. Two major dietary patterns were extracted, labelled the Western and Prudent patterns, explaining 10.9% and 8.0% of the total variance in dietary intake, respectively; Table 1 shows the factor loadings for these patterns. The dietary pattern score was constructed by summing intakes of the food groups, weighted by factor loadings, with all participants having Western Diet and Prudent Diet scores constructed.

### 2.3. [^18^F]-FDG-PET Imaging

[^18^F]-FDG-PET was performed at baseline in all participants (n = 132). Assessments were conducted at discrete timepoints according to the study protocol, with approximately 18-month intervals between visits; however, follow-up participation varied across individuals due to scan availability. Participants were scheduled to undergo follow-up assessments at approximately 18-month intervals. Among participants with at least one follow-up FDG-PET scan, the mean follow-up duration was 6.3 months (Standard Deviation = 9.24), (range: 0–43 months), indicating substantial variation in follow-up duration across individuals. Overall, 69 participants completed one additional scan (a total of two scans), while 4 participants completed two additional scans (a total of three scans), over a maximum follow-up period of 43 months, using standard brain imaging protocols on a GSO Philips Allegro PET camera, as reported previously [20]. Participants fasted for at least 4 hours prior to tracer administration. Subsequently, approximately 185 megabecquerel (MBq; 5–7 MBq/kg) FDG was administered via a peripheral intravenous cannula while the subjects rested in a quiet room. Imaging commenced 45 minutes post-isotope administration. Emission and transmission images were obtained over a 20 min period.

Quantitative analysis of [^18^F]-FDG-PET images was performed using the CapAIBL PET-only approach [21,22]. The retention of [^18^F]-FDG was quantified using a standardized uptake value ratio (SUVR), which represents tracer uptake divided by the average uptake in the pons as the reference region [23,24]. The SUVR was computed for neocortex as the mean SUVR in the neocortical grey matter (GM) region, which comprised the frontal, superior parietal, lateral temporal, occipital, anterior, and posterior cingulate regions [24]. The SUVR retention was also analyzed independently in cortical regions of interest (ROIs), including the dorsolateral prefrontal, ventrolateral prefrontal, orbitofrontal, gyri rectus, temporo-occipital, temporal, inferior temporal, amygdala, hippocampus, parahippocampus, fusiform, supramarginal gyrus, angular gyrus, entorhinal, superior parietal, and posterior cingulate regions. The definition of these ROIs is based on the Automated Anatomical Labelling (AAL) [25]. The SUVRs for the dorsolateral prefrontal, ventrolateral prefrontal, orbitofrontal, and gyrus rectus regions were summed to form an overall score for the frontal cortex. The SUVRs for the temporo-occipital, temporal, inferior temporal, amygdala, hippocampus, parahippocampus, fusiform, and entorhinal regions were summed to form an overall score for the temporal cortex. The SUVRs for the supramarginal and angular gyri were summed to form an overall score for the inferior parietal region.

The pons was chosen as the reference region [23,24] because it exhibits lower variability across individuals and more stable uptake values, reinforcing its suitability for reducing overall noise in SUVR calculations and enhancing analytic sensitivity [26].

### 2.4. Apolipoprotein E Genotyping

Fasting blood samples obtained using standard venipuncture of the antecubital vein were collected into Ethylenediaminetetraacetic acid (EDTA) tubes containing Prostaglandin E1 (Sapphire Biosciences (Redfern, NSW, Australia), 33·3 Nanogram/millilitre (ng/mL)) to prevent platelet activation. Whole blood was centrifuged to separate leucocytes. Deoxyribonucleic acid (DNA) was isolated from the leucocytes, using Qiagen Midiprep kits (Qiagen, Hilden, Germany). The APOE genotype was subsequently determined using polymerase chain reaction amplification and restriction enzyme digest techniques [26]. Apolipoprotein E (APOE) ε4 carriage is the strongest common genetic risk factor for late-onset Alzheimer’s disease and has been associated with increased amyloid deposition and early cerebral glucose hypometabolism, even in cognitively normal individuals. Given its established role in Alzheimer’s disease risk stratification and its potential influence on brain metabolic trajectories, APOE ε4 status was included as a covariate in all statistical models.

### 2.5. Statistical Analysis

All statistical analyses were performed using R version 4.3.2 for Windows. A *p*-value of 0.05 or smaller determined a significant result. Means, standard deviations, and percentages are provided in Table 2 for the entire cohort and for stratification by sex. Independent-sample *t*-tests and chi-square (χ^2^) analyses were conducted to evaluate group differences, as appropriate.

To assess the cohort for cognitive impairment, a cut-off score of <23 on the Montreal Cognitive Assessment (MoCA), (R (Programming Language), Version: 4.3.2; Manufacturer: R Foundation for Statistical Computing, Vienna, Austria) was utilized as a distinction between unimpaired cognition and mild cognitive impairment [27,28,29]. Independent-sample *t*-tests and χ^2^ analyses evaluated group differences between those with <23 MoCA scores and those with ≥23 MoCA scores.

A linear mixed-effects model was fitted to analyze the association between FDG-PET SUVR (dependent variable) and baseline dietary pattern (independent variable) [30]. Models used a random intercept by ID (which considers the multiple observations by each individual for the longitudinal data), with age, sex, energy intake, body mass index (BMI), a time from CCVFFQ data collection to baseline FDG-PET date as these varied between participants, and APOE genotype (absence of ε4 allele vs. presence of either one or two ε4 alleles) included as independent variables. Food groups were entered as absolute intakes (g/day) without prior energy adjustment. Total energy intake was included as a covariate in the regression models. This model was fitted in R using the package ‘lmerTest’ [31]. This analysis was repeated following stratification of the cohort by sex. The False Discovery Rate (FDR) correction using the Benjamini–Hochberg procedure was implemented in R (p.adjust, method = “fdr”). FDR adjustment was performed separately within each linear mixed model, consistent with standard practice in which correction is applied to the set of simultaneously tested hypotheses within a given analysis [32].

## 3. Results

### 3.1. Demographics

This cohort comprised participants (n = 132) from the WAMS (38.6% male) with an average age of 70.4 ± 5.9 years. Participants had an average BMI of 26.9 ± 4.3 kg/m^2^, and 34.8% were carriers of the APOE ε4 allele (Table 2). When the cohort was stratified by sex, as expected, males had higher energy intake (*p* = 0.007) and a higher Western Diet score (*p* < 0.001) than females.

Fourteen individuals scored below the 23-point MoCA cut-off. Independent-sample *t*-tests and χ^2^ analyses evaluated group differences between those with <23 MoCA scores and those with ≥23 MoCA scores. Baseline comparisons between groups (<23 and ≥23 MoCA scores) revealed no significant differences in energy intake, age, BMI, Western Diet score, or Prudent Diet score. However, there was a significant difference in the proportion of females to males (*p* = 0.041), with the <23 MoCA scoring group having a higher percentage of males (64.3% vs. 36.1%).

### 3.2. Linear Mixed Effects Models

Linear mixed-effect models were used to analyze the association between Prudent and Western dietary adherence and FDG-PET SUVR.

No associations were observed between dietary patterns and FDG-PET SUVR in the cohort as a whole (Table 3).

In females, following FDR adjustment, Western Diet score was negatively associated with glucose metabolism in the left fusiform gyrus (standardized β = −0.00062; SE = 0.00025; FDR adjusted *p* = 0.043), neocortex (standardized β = −0.00063; SE = 0.00026; FDR adjusted *p* = 0.047), left ventrolateral prefrontal (standardized β = −0.00083; SE = 0.00032; FDR adjusted *p* = 0.045 and inferior parietal region (standardized β = −0.00344; SE = 0.00129; FDR adjusted *p* = 0.033; Table 4).

No associations were observed between dietary patterns and FDG-PET SUVR in males.

## 4. Discussion

Our study aimed to explore the impact of two ‘a posteriori’ dietary patterns—the Western Diet (an ‘unhealthy’ dietary pattern) and the Prudent Diet (a ‘healthy’ dietary pattern)—on FDG uptake at baseline and at up to two follow-up assessments scheduled approximately 18 months apart, over a maximum follow-up period of 43 months. We observed that in females, higher Western Diet adherence at baseline was associated with faster reductions in FDG uptake, specifically in the left fusiform gyrus, neocortex, left ventrolateral prefrontal, and inferior parietal region. No associations were observed between dietary patterns and FDG uptake in the cohort as a whole or in males. Given the observational nature of this study, the findings should be interpreted as associations and do not imply causality.

Additionally, the study observed statistically significant sex differences between MoCA groups (scoring < 23 and ≥23; *p* = 0.041), with a higher proportion of males in the <23 group (64.3% vs. 36.1%). This finding aligns with prior evidence suggesting demographic variations in cognitive screening outcomes, highlighting the potential role of sex-specific factors in cognitive performance and test sensitivity [33]. Future studies could further explore these differences to clarify underlying mechanisms. Furthermore, a sensitivity analysis excluding participants with MoCA scores < 23 was not performed. Although this subgroup was small (n = 14), their inclusion may have introduced heterogeneity. Future studies should assess whether associations remain consistent after excluding individuals with potential cognitive impairment.

Our findings suggest a modest negative impact of consuming an unhealthy diet on brain glucose metabolism in females over time. There is a paucity of previous studies investigating dietary patterns and glucose metabolism. In one of the few studies available on this topic, Berti et al. observed that a ‘fatty’ dietary pattern, similar to our Western Diet pattern, was negatively associated with glucose metabolism in the middle and inferior temporal cortex (bilaterally), the right frontal cortex, and the left parietal cortex, as measured with FDG-PET (*p* < 0.001) [34]. The authors concluded that diet could be modulating AD-risk through its effects on Aβ and associated neuronal impairment, which exacerbates neuroinflammation and oxidative stress, further contributing to neuronal damage. Overall, while evidence links unhealthy dietary patterns to metabolic dysregulation in the brain, further intervention studies are warranted to elucidate causality and potential mechanisms.

Research has shown that dietary patterns aligned with the Western Diet pattern negatively impact critical metabolic pathways, such as the tricarboxylic acid (TCA) cycle and β-oxidation, both essential for energy production in neural tissues [35]. Mice fed a Western Diet exhibit elevated levels of lactate and lipids in the brain, suggesting that the normal metabolic processes are impaired. These alterations in brain metabolism may contribute to an overall reduction in energy availability for neuronal functions, which is essential for maintaining cognitive health and preventing neurodegeneration [35].

Our findings did not demonstrate significant associations between the self-reported Prudent Diet and FDG PET SUVR. This agrees with previous evidence and suggests that a Prudent Diet might not have a significant impact on cerebral glucose metabolism [36,37]. No significant associations were observed between the Prudent dietary pattern and FDG-PET SUVR, suggesting that, within this cohort, adherence to this dietary pattern was not associated with changes in cerebral glucose metabolism. While this study refers to its dietary pattern as the Prudent Diet, the specific components—dark-yellow vegetables, cruciferous vegetables, garlic, green leafy vegetables, fruits, other vegetables, and whole grains—differ from those in other studies using the same term. In many instances, the Prudent Diet is described more generally, emphasizing plant-based foods, whole grains, and low-fat dairy, with reduced intakes of processed foods and red meat [38]. For example, a study by Fung et al., which explored dietary patterns in U.S. populations, characterized the Prudent Diet as high in fruits, vegetables, legumes, fish, and whole grains, without further subdivision of vegetable types or mention of garlic [38]. Similarly, another trial focused on cardiovascular health utilized a Prudent Diet rich in plant-based foods but did not distinguish between vegetable subcategories such as cruciferous or dark-yellow varieties [39]. These broader definitions contrast with our study’s more detailed categorization, which incorporates nutrient-dense foods known for their anti-inflammatory and antioxidant properties, such as allicin in garlic and glucosinolates in cruciferous vegetables [39]. This distinction highlights variability in the term Prudent Diet and highlights the importance of detailed dietary descriptions in research. These differences illustrate that the term Prudent Diet serves as a conceptual framework rather than a standardized dietary protocol, necessitating caution when comparing findings across studies, and providing a possible reason for divergent results.

In healthy ageing individuals, a brain glucose deficit occurs primarily in the frontal cortex as the brain’s ability to utilize glucose for energy declines with age, whereas in AD, the parietal and temporal lobes are adversely affected [40]. Our findings observed glucose hypometabolism in frontal (ventrolateral prefrontal cortex), parietal (inferior parietal), and temporal (left fusiform) lobes, as well as the neocortex, the outermost layer of the brain covering the frontal, parietal, occipital, and temporal lobes. Whilst the ventrolateral prefrontal cortex is involved in executive control and emotion regulation and may be especially vulnerable to dietary influences that impair metabolic function and self-regulatory processes [41], the ventrolateral prefrontal cortex is known to be particularly vulnerable to age-related metabolic decline; however, the observed association with Western dietary pattern adherence suggests that dietary factors may contribute to or exacerbate this vulnerability. The fusiform gyrus is a critical brain region situated at the intersection of the temporal and occipital lobes, playing a pivotal role in various cognitive and perceptual processes. The main functions are visual processing, particularly facial recognition, which helps differentiate faces and recognize individuals [42]. It is also involved in high-level object recognition, such as identifying words, numbers, or objects, making it essential for tasks that require distinguishing between similar visual stimuli [42]. The fusiform gyrus has been shown to exhibit significant metabolic decline in AD, with previous FDG-PET studies revealing that patients with AD demonstrate marked reductions in glucose metabolism in the fusiform gyrus compared to cognitively unimpaired controls. This metabolic decline correlates with the characteristic cognitive and functional impairments seen in AD, such as difficulties in visual-spatial processing and facial recognition [42]. Additionally, the fusiform, along with the inferior temporal cortex, is the first region affected by cortical tau when it spreads out of the medial temporal lobe [43], providing another example of the importance of this region in the early stages of the disease process and an explanation as to why the observed low FDG signal was not unexpected in this region. This hypometabolism in the fusiform gyrus is considered a sensitive marker of disease progression, potentially appearing in the early and prodromal stages of AD, including mild cognitive impairment (MCI) [6].

Additionally, our results show a negative association between adherence to the Western Diet and neocortex glucose metabolism in female participants. The neocortex is critical for higher brain processes, including perception, learning, memory, and goal-directed behaviour [44], with the neocortex being a main area affected by AD pathology early in the disease process [45]. FDG-PET has revealed that one of the earliest and most characteristic neuroimaging biomarkers of AD is hypometabolism in the neocortex, particularly in the posterior cingulate cortex, precuneus, and inferior parietal lobule [46]. These regions are highly metabolically active in healthy individuals, but in AD, they show significant reductions in glucose uptake even at preclinical stages, correlating with Aβ burden and tau pathology [46]. Cortex hypometabolism is exacerbated by systemic metabolic disturbances, such as insulin resistance and neuroinflammation, often linked to diets high in saturated fats, refined sugars, and processed foods [47]. Furthermore, higher adherence to the Western Diet was associated with decreased glucose metabolism in the inferior parietal lobule, a region responsible for sensory integration, visuospatial processing, and attention, which are also commonly affected in early AD [48,49].

Our findings were observed in females with no significant associations in males, although males in our cohort had a significantly higher Western Diet score at baseline than females. Females not only represent nearly two-thirds of AD cases but also appear to exhibit heightened vulnerability to key pathological mechanisms, including greater susceptibility to mitochondrial dysfunction, synaptic loss, and tau accumulation [50]. As highlighted by Podcasy and Epperson [50], these biological and hormonal factors—especially those associated with estrogen decline—may amplify the detrimental effects of metabolic disturbances induced by poor dietary intake. However, as these variables were not directly measured in this present study, these interpretations remain speculative and require confirmation in future studies. This suggests that Western Diets, which promote neuroinflammation and insulin resistance, could exert a disproportionately negative impact on cerebral metabolism in females. Considering sex as a biological variable is therefore essential when interpreting dietary influences on neurodegenerative processes [50].

The current study capitalizes on the well-defined dataset from the Western Australian Memory Study; however, the analysis is not without limitations. Firstly, the sample size of 132 cognitively unimpaired participants may have limited the ability to detect associations. Moreover, dietary intake was assessed using the CCVFFQ, which relies on self-reported data and may be subject to recall bias and measurement error. Dietary intake was assessed only at baseline using the CCVFFQ, which captures habitual intake over the previous 12 months but does not account for potential changes in diet over time. As dietary behaviours may vary during the 43-month follow-up, this represents a key limitation and may have introduced exposure misclassification. However, such misclassification is likely to be non-differential and would bias results toward the null, potentially underestimating true associations. Future studies should consider larger, more diverse cohorts, use objective dietary monitoring methods to enhance accuracy, and conduct repeated dietary assessments to better characterize longitudinal dietary patterns and their impact on brain metabolism. Future research should consider larger, more diverse cohorts and utilize objective dietary monitoring methods to enhance accuracy. A key limitation of this study is the absence of structural MRI data to account for cortical atrophy and partial-volume effects. Reduced FDG-PET SUVR in regions affected by neurodegeneration may reflect, in part, reduced tissue volume rather than true metabolic decline. Although FDG-PET hypometabolism is a well-established marker of neuronal dysfunction, the inability to adjust for cortical atrophy limits the interpretation of these findings. Future studies incorporating multimodal imaging approaches, including MRI-based measures of cortical thickness and partial-volume correction, are needed to better distinguish between structural and metabolic contributions to observed changes in glucose metabolism. The sex-specific findings observed in this study should also be interpreted with caution. Although analyses were stratified by sex, the models did not account for several potentially important sex-related and lifestyle factors, including physical activity, menopausal status, hormone replacement therapy, and cardiovascular medication use. These variables may differ between males and females and are known to influence brain metabolism and neurodegenerative risk. As such, residual confounding may have contributed to the observed associations. Future studies incorporating more comprehensive covariate adjustment are needed to clarify whether these sex differences reflect true biological effects or unmeasured confounding. Additionally, future research should consider randomized controlled trials to establish robust temporal and causal relationships. These studies can enhance the validity of findings by minimizing confounding variables and providing stronger evidence for cause-and-effect mechanisms, thereby contributing to a deeper and more reliable understanding of the relationship between diet and brain glucose metabolism. Increasing the follow-up period could expand understanding of dietary effects on glucose metabolism, helping clarify the relationship between dietary patterns and cognitive health.

## 5. Conclusions

The relationship between dietary patterns and cognitive health is increasingly recognized as crucial, particularly in the context of AD and other neurodegenerative disorders. Our study suggests that higher adherence to a Western dietary pattern, characterized by high sugar and saturated fat intake, is associated with reduced brain glucose metabolism, particularly among individuals with a higher risk for AD due to the female sex. A greater decline in glucose metabolism was observed in the left fusiform gyrus, neocortex, left ventrolateral prefrontal cortex, and inferior parietal region. Therefore, progressive glucose hypometabolism could serve as a biomarker for neurodegeneration and highlights the potential for dietary interventions to mitigate cognitive decline. No significant associations were observed between the Prudent dietary pattern and brain glucose metabolism. Therefore, no conclusions can be drawn regarding its potential protective role in this cohort. Future research should focus on elucidating the mechanisms by which diet influences brain function and glucose metabolism, and on exploring the potential of dietary interventions as preventive strategies against neurodegenerative diseases such as AD.

## Figures and Tables

**Table 1:** Factor loadings for the two major factors (diet patterns) identified from principal components analysis.

	Western Diet	Prudent Diet
Red meats	0.727	
Processed meats	0.614	
Chips	0.566	
Sweets	0.536	
Poultry	0.517	
Refined grains	0.512	
Condiments	0.449	
Potatoes	0.401	
Other breakfast cereals	0.358	
Beer	0.390	
Fish	0.335	
Meat pies	0.333	
Pizza	0.333	
Fruit juice	0.301	
Other vegetables		0.749
Dark-yellow vegetables		0.669
Green leafy vegetables		0.531
Fruit		0.505
Cruciferous vegetables		0.461
Garlic		0.405
Whole grains		0.361

**Table 2:** Descriptive statistics for the cognitively unimpaired cohort who completed the CCVFFQ, and subgroups of the cohort based on stratification by sex.

	Total Sample (n = 132)	Standard Deviation	Female (n = 81)	Standard Deviation	Male (n = 51)	Standard Deviation	*p*-Values for Sex Differences
Age, y	70.4	5.9	70.05	5.9	71.04	6.0	0.357
Sex, males; n (%)	51 (38.6)						
Presence of APOE ε4 allele; n (%)	46 (34.8)		23 (28.4)		23 (45.1)		0.050
Energy Kcal	1634.2	563.9	1530.3	549.4	1799.3	552.0	**0.007**
Baseline body mass index *, kg/m^2^	26.9	4.3	26.90	4.4	27.05	4.2	0.854
Baseline Western Diet score	199.9	138.3	158.02	92.4	266.3	179.7	**<0.001**
Baseline Prudent Diet score	227.0	102.4	238.3	106.8	209.0	93.2	0.100

Abbreviations: APOE, apolipoprotein E; CCVFFQ, Cancer Council of Victoria Food Frequency Questionnaire; kg, kilogram; m^2^, metre squared; y, years. Bold indicates statistical significance (*p* < 0.05). Characteristics compared using an independent-sample *t*-test for continuous variables and a χ^2^ test for categorical variables. * Body Mass Index is calculated as weight in kilograms divided by height in metres.

**Table 3:** Results of linear mixed effect model analyses examining the association between baseline diet scores and change in FDG-PET SUVR over 43 months. Standardized β values are shown.

	Western Diet				Prudent Diet			
Brain Region	Β	SE	*p*-Value	FDR Adjusted *p*-Value	β	SE	*p*-Value	FDR Adjusted ***p***-Value
Neocortex	−0.00011	0.00011	0.309	0.392	0.00004	0.00010	0.710	0.818
L Dorsolateral Prefrontal	−0.00017	0.00015	0.252	0.370	0.00010	0.00014	0.466	0.622
R Dorsolateral Prefrontal	−0.00015	0.00015	0.338	0.452	0.00008	0.00014	0.559	0.745
L Ventrolateral Prefrontal	−0.00021	0.00013	0.117	0.187	0.00008	0.00012	0.513	0.681
R Ventrolateral Prefrontal	−0.00019	0.00013	0.154	0.246	0.00004	0.00012	0.744	0.744
L Orbito Frontal	−0.00021	0.00013	0.112	0.179	0.00008	0.00012	0.517	0.689
R Orbito Frontal	−0.00021	0.00013	0.108	0.216	0.00005	0.00012	0.685	0.779
L Gyrus Rectus	−0.00016	0.00011	0.157	0.264	−0.00004	0.00010	0.675	0.771
R Gyrus Rectus	−0.00017	0.00012	0.151	0.302	−0.00004	0.00011	0.723	0.827
Frontal Cortex	−0.00147	0.00098	0.137	0.220	0.00035	0.00090	0.702	0.936
Inferior Parietal Region	−0.00037	0.00052	0.478	0.546	0.00010	0.00047	0.835	0.923
L Temporal	−0.00016	0.00010	0.115	0.153	0.00009	0.00009	0.327	0.457
R Temporal	−0.00010	0.00011	0.364	0.364	0.00002	0.00010	0.878	0.878
L Amygdala	−0.00002	0.00008	0.768	0.877	−0.00006	0.00007	0.358	0.637
R Amygdala	0.00007	0.00008	0.369	0.639	−0.00005	0.00007	0.509	0.958
L Hippocampus	−0.00006	0.00006	0.351	0.562	0.00005	0.00006	0.387	0.773
R Hippocampus	0.00004	0.00007	0.581	0.785	0.00004	0.00006	0.553	0.841
L Parahippocampus	−0.00007	0.00007	0.338	0.451	0.00001	0.00006	0.858	0.981
R Parahippocampus	−0.00005	0.00006	0.466	0.664	−0.00004	0.00006	0.534	0.861
L Entorhinal	0.00008	0.00015	0.584	0.664	−0.00018	0.00013	0.161	0.384
R Entorhinal	0.00004	0.00012	0.753	0.802	−0.00009	0.00010	0.375	0.600
L Fusiform	−0.00014	0.00010	0.143	0.190	0.00006	0.00009	0.524	0.524
R Fusiform	−0.00004	0.00009	0.664	0.757	−0.00003	0.00009	0.708	0.860
Temporal Cortex	−0.00047	0.00104	0.653	0.715	−0.00026	0.00095	0.787	0.787

Abbreviations: APOE, apolipoprotein E; FDG-PET, [^18^F]-Fluorodeoxyglucose positron emission tomography; FDR, false discovery rate; L, left; R, right; SE, standard error; SUVR, standardized uptake value ratio. The model includes age, APOE ε4 allele carriage, sex, energy intake, and body mass index as independent variables.

**Table 4:** Results of linear mixed effect model analyses examining the association between baseline diet scores and change in FDG PET SUVR over 43 months, stratified by sex. Standardized β values are shown.

	Western Diet					Prudent Diet			
Brain Region	Sex	Β	SE	*p*-Value	FDR Adjusted *p*-Value	Β	SE	*p*-Value	FDR Adjusted ***p***-Value
Neocortex	Female	−0.00063	0.00027	0.020 *	0.047	0.00000	0.00012	0.999	0.999
L Dorsolateral Prefrontal	Female	−0.00080	0.00040	0.049	0.115	0.00002	0.00018	0.910	0.910
R Dorsolateral Prefrontal	Female	−0.00085	0.00038	0.029	0.068	−0.00008	0.00017	0.658	0.836
L Ventrolateral Prefrontal	Female	−0.00083	0.00032	0.013 *	0.045	0.00006	0.00015	0.700	0.723
R Ventrolateral Prefrontal	Female	−0.00075	0.00033	0.027	0.094	0.00002	0.00015	0.913	0.971
L Orbito Frontal	Female	−0.00056	0.00033	0.092	0.162	0.00004	0.00015	0.769	0.769
R Orbito Frontal	Female	−0.00060	0.00033	0.070	0.164	0.00002	0.00014	0.899	0.946
L Gyrus Rectus	Female	−0.00050	0.00029	0.089	0.177	−0.00007	0.00013	0.584	0.681
R Gyrus Rectus	Female	−0.00050	0.00030	0.102	0.237	−0.00007	0.00013	0.574	0.804
Frontal Cortex	Female	−0.00539	0.00247	0.032	0.109	−0.00007	0.00110	0.952	0.972
Inferior Parietal Region	Female	−0.00344	0.00129	0.010 **	0.033	−0.00005	0.00059	0.934	0.934
L Temporal	Female	−0.00057	0.00026	0.031	0.059	0.00010	0.00012	0.406	0.711
R Temporal	Female	−0.00058	0.00026	0.028	0.091	0.00002	0.00012	0.894	0.972
L Amygdala	Female	−0.00007	0.00021	0.748	0.932	−0.00008	0.00009	0.387	0.578
R Amygdala	Female	0.00008	0.00021	0.715	0.919	−0.00006	0.00009	0.549	0.902
L Hippocampus	Female	−0.00017	0.00017	0.315	0.509	0.00002	0.00007	0.817	0.975
R Hippocampus	Female	−0.00015	0.00019	0.410	0.945	0.00006	0.00008	0.467	0.945
L Parahippocampus	Female	−0.00003	0.00018	0.875	0.875	0.00000	0.00008	0.964	0.964
R Parahippocampus	Female	−0.00018	0.00017	0.292	0.560	−0.00006	0.00007	0.422	0.738
L Entorhinal	Female	−0.00012	0.00038	0.761	0.793	−0.00017	0.00016	0.310	0.724
R Entorhinal	Female	−0.00027	0.00029	0.353	0.617	−0.00002	0.00012	0.863	0.863
L Fusiform	Female	−0.00063	0.00025	0.016 *	0.043	0.00010	0.00011	0.391	0.547
R Fusiform	Female	−0.00039	0.00024	0.116	0.225	0.00003	0.00011	0.747	0.871
Neocortex	Male	−0.00001	0.00013	0.958	0.958	0.00008	0.00020	0.682	0.797
L Dorsolateral Prefrontal	Male	−0.00001	0.00017	0.971	0.971	0.00020	0.00025	0.436	0.763
R Dorsolateral Prefrontal	Male	0.00004	0.00019	0.843	0.889	0.00038	0.00028	0.184	0.321
L Ventrolateral Prefrontal	Male	−0.00007	0.00017	0.704	0.919	0.00007	0.00026	0.787	0.837
R Ventrolateral Prefrontal	Male	−0.00010	0.00017	0.574	0.882	0.00008	0.00025	0.748	0.826
L Orbito Frontal	Male	−0.00009	0.00015	0.561	0.856	0.00008	0.00023	0.719	0.839
R Orbito Frontal	Male	−0.00014	0.00015	0.369	0.645	0.00008	0.00023	0.741	0.746
L Gyrus Rectus	Male	−0.00010	0.00014	0.505	0.687	0.00001	0.00021	0.976	0.976
R Gyrus Rectus	Male	−0.00014	0.00014	0.347	0.607	0.00006	0.00021	0.782	0.936
Frontal Cortex	Male	−0.00059	0.00122	0.632	0.860	0.00095	0.00181	0.602	0.880
Inferior Parietal Region	Male	0.00025	0.00062	0.692	0.807	0.00017	0.00091	0.853	0.853
L Temporal	Male	−0.00009	0.00012	0.442	0.515	0.00002	0.00017	0.923	0.923
R Temporal	Male	−0.00004	0.00014	0.756	0.756	−0.00003	0.00020	0.896	0.896
L Amygdala	Male	0.00002	0.00009	0.801	0.831	−0.00013	0.00013	0.311	0.725
R Amygdala	Male	0.00008	0.00009	0.383	0.868	−0.00009	0.00014	0.518	0.737
L Hippocampus	Male	−0.00003	0.00007	0.691	0.767	0.00008	0.00011	0.431	0.755
R Hippocampus	Male	0.00008	0.00008	0.321	0.561	−0.00002	0.00012	0.863	0.863
L Parahippocampus	Male	−0.00006	0.00009	0.491	0.622	−0.00001	0.00013	0.962	0.962
R Parahippocampus	Male	−0.00006	0.00008	0.416	0.519	0.00003	0.00011	0.793	0.866
Parahippocampus	Male	−0.00012	0.00015	0.430	0.543	0.00003	0.00023	0.911	0.936
L Entorhinal	Male	0.00009	0.00017	0.599	0.907	−0.00018	0.00025	0.488	0.854
R Entorhinal	Male	0.00011	0.00015	0.480	0.840	−0.00029	0.00022	0.192	0.449
L Fusiform	Male	−0.00013	0.00011	0.232	0.271	−0.00001	0.00016	0.960	0.960
R Fusiform	Male	−0.00004	0.00011	0.732	0.732	−0.00019	0.00016	0.246	0.287
Temporal Cortex	Male	−0.00001	0.00124	0.996	0.996	−0.00096	0.00182	0.601	0.601

Abbreviations: APOE, apolipoprotein E; FDG-PET, [^18^F]-Fluorodeoxyglucose positron emission tomography; FDR, false discovery rate; L, left; R, right; SE, standard error; SUVR, standardized uptake value ratio. The model includes age, APOE ε4 allele carriage, energy intake, and body mass index as independent variables. Bold indicates statistical significance (* FDR adjusted *p* < 0.05; ** FDR adjusted *p* < 0.01).

## Data Availability

The data presented in this study are not publicly available due to privacy and ethical restrictions, as well as ongoing longitudinal data collection within the Western Australian Memory Study. De-identified data may be made available from the corresponding author upon reasonable request and subject to institutional and ethics approval.

## Abbreviations

AAL	Automated Anatomical Labelling
AD	Alzheimer’s Disease
Aβ	Beta-amyloid
APOE	Apolipoprotein E
BMI	Body Mass Index
CCVFFQ	Cancer Council of Victoria Food Frequency Questionnaire
DNA	Deoxyribonucleic Acid
EDTA	Ethylenediaminetetraacetic Acid
FDG	[^18^F]-Fluorodeoxyglucose
FDG-PET	[^18^F]-Fluorodeoxyglucose Positron Emission Tomography
FDR	False Discovery Rate
FFQ	Food Frequency Questionnaire
GLUT3	Glucose Transporter 3
GM	Grey Matter
IDE	Insulin-degrading Enzyme
IRS-2	Insulin Receptor Substrate 2
MBq	Megabecquerel
MCI	Mild Cognitive Impairment
MoCA	Montreal Cognitive Assessment
MRI	Magnetic Resonance Imaging
NHMRC	National Health and Medical Research Council
PET	Positron Emission Tomography
PUFAs	Polyunsaturated Fatty Acids
rCMRglc	Regional Cerebral Metabolic Rate of Glucose
ROI	Region of Interest
SE	Standard Error
SPM	Statistical Parametric Mapping
SUVR	Standardized Uptake Value Ratio
TCA	Tricarboxylic Acid
WAMS	Western Australian Memory Study
